# Notes on Obstetrics and Gynæcology

**Published:** 1875-07

**Authors:** E. N. Brush


### MEDICAL NOTES.

ART. I.—*Notes on Obstetrics and Gynæcology.* By E. N. BRUSH, M. D.

1. Three Cases of Successful Removal of Fibro-Cystic Tumors of the Uterus. By THOMAS KEITH, M. D., F.R.C.S. (*London Lancet*, July, 1875.)

2. A Novel Method of Treating the Vomiting of Pregnancy. By EDWARD COPEMAN, M. D., etc. (*British Medical Journal*, May 15th, 1875.) (*The Medical Record*, July 17, 1875.)

3. Quinia as a Stimulant to the Pregnant Uterus. By ALBERT H. SMITH, M. D. (*Obstet. Journal Gt. Britain and Ireland. Am. Supplement*, June, 1875.)

4. A Clinical Contribution to the Treatment of Tubal Pregnancy. By T. GAILLARD THOMAS, M. D. (*New York Medical Journal*, June, 1875.)

5. A Case of Hirsuties Gestationis. By CHARLES E. SLOCUM, M. D. (*Medical Record*, July 10, 1875.)

1. The three cases embodied in this report are the only ones in which Dr. Kieth has interfered with uterine tumors by abdominal section, and from his experience, he concludes that the operation is not one to be lightly undertaken.

Case I was operated upon under the impression that it was ovarian in character. The patient was an unmarried lady aged 52. The tumor was first discovered by the patient in Dec., 1872. It was then pronounced by Dr. Perry of Glasgow, a fibrous tumor of the uterus. The tumor rapidly enlarged and in six months from her first consultation with Dr. Perry, was distinctly cystic in character and reached above the umbilicus.

A cursory examination in October, 1874, convinced Dr. Kieth that it was ovarian in character, and on the 2d of November, the operation for its removal was undertaken.

Upon opening the abdominal cavity and tapping the cyst, the error in diagnosis was made manifest. The tumor together with the left ovary was removed, the stump being secured by a steel wire. Thirty-seven days after the operation the patient was discharged cured and returned to Glasgow.

The error in this case was not at all unexceptionable, but Dr. Kieth says that it is the first mistaken diagnosis he has made in one hundred and ninety-four cases.

Case II was also an unmarried woman 44 years of age. The operation was made in December last. The operation was in all respects like the first one. The stump was included in steel wire and left in the lower margin of the wound. In this case the adhesions were extensive, and were only broken up after considerable effort. Several bleeding points were left from which the hemorrhage was quite free. Drainage was kept up by a glass tube. In six weeks the patient was sent home well.

Case III. This case was one of rapid growth, accompanied by pain and profuse hemorrhage. The patient, aged forty, was sent to Dr. Kieth, from Londonderry.

The uterus and both ovaries, together with numerous cysts of the broad ligament were removed. This case had a profuse secondary hemorrhage seven days after the operation, which was however promptly checked.

The advice which Dr. Kieth has hitherto given in the numerous cases of uterine tumors which have passed through his hands has been to let them alone.

He now expresses the hope that the time is not far distant when many of these unfortunate patients may look to surgery with as much hope of relief as those afflicted with ovarian disease now do.

2. The cases which Dr. Copeman has reported under this head are three in number. The first was six months advanced in pregnancy when she was seen by Dr. Copeman in consultation.

The vomiting was so excessive that fears were entertained for her safety. Dr. Copeman advised the induction of premature labor, to which the others were at first unwilling to agree, but finally consented. He at once dilated the os uteri with his finger so that the membranes could be felt. An attempt was then made to rupture them, but on account of their flaccid condition and the lack of a proper instrument it failed. In an hour the patient was seen again, and it was found that vomiting had not recurred. It was now decided to await developements. Nourishment was administered and the case watched. Vomiting did not again occur and the patient progressed favorably to the full term.

In the second case, one of two months standing, the physician had tried all the known remedies, but without success. Dr. Copeman was called, but remembering the first case, he dilated the os as much as he could with his finger, passing it all around and removing the puckering making its edge smooth. The patient vomited but once after the procedure, and at last accounts her confinement was shortly anticipated.

In the third case the patient had previously been confined nine times, and generally during the early months of pregnancy, and sometimes for several months, she had been troubled with vomiting. In this pregnancy the sickness had been most constant for three weeks. Nothing could be retained on the stomach and she was in a very weak condition. There was no dropsy, but considerable albumen, some pus, and casts were found in the urine. On examination the os was found puckered and dilatable. He dilated it at once as in the other cases. In a few days the sickness had entirely passed off and she was able to retain food. In due course of time the patient was safely delivered and is doing well.

Dr. Copeman does not attempt an explanation of the *modus operandi* of these cases, but presents them for consideration.

3. Dr. Smith says: "Having for several years, and more carefully during the last few months, noticed the action of quinia during the various periods of pregnancy and parturition, and having been enabled to draw some practical conclusions of no little value to myself, I have thought that perhaps a brief statement of my results might be of interest."

He says that having had on several occasions patients under his care, suffering from malarial disorders who were at the time pregnant, he has administered quinia in doses varying from twelve to twenty grains in the twenty-four hours, without the slightest manifestation of disturbance of the uterus. In cases also in which accompanying the malarial disorder were pelvic distress, tenesmus and sacro-lumbar pains, the administrarion of large doses of quinia quieted them.

Of the effects of quinia upon the prematurely contracting uterus, the experience of the writer is too limited to allow him to speak with any definiteness. In five cases however, fifteen grains were administered to each after the process had advanced beyond arrest, but the effect upon the contractions or hemorrhage was wholly inert. These trials if conclusive would forbid placing quinia with ergot among the excito-motor stimulants with specific effect upon the uterine muscular fibre.

Of quinia as an aid to natural parturition from Dr. Smith's observations more may be expected. During the past few months he has been carefully watching its effects. He has made observations upon forty-two cases such as occurred in the ordinary run of practice. To each of these he administered as soon as actual labor pains commenced, fifteen grains of the sulphate of quinia, in one dose. In every case he observed within fifteen minutes a decided increase in the frequency of the pains, a rapid progress, and where no obstructions existed a speedy termination. The conclusions which he arrives at in regard to the action of the drug in these cases are as follows:

It increases the activity of the normal uterine contractions; the pains becoming more frequent and more intense, the expulsive power being greater, while the yielding of the circular fibres of the os is more prompt; the contractions maintaining their proper inmittent character, the relaxation and rest in the interval being complete; showing in this respect an entirely different action from the continuous spasmodic contraction caused by ergot. The efficiency of the the contraction may be judged of from the fact, that, in the thirty-two cases having no obstruction, although many were primiparæ, and a larger than usual proportion occipito-posterior positions, the average duration of active labor after the quinia was administered was about one hour. In a considerable number of the cases included, I had in several previous labors required to use forceps to combat inertia in the second stage.

It promotes permanent tonic contraction of the uterus, after the expulsion of the placenta. Several of the patients had had flooding under my care previously, some of them habitually, and some stated they had always had a profuse and weakening flow in their other labors. In the whole forty-two I had not one case of flooding, and as a rule the uterus contracted firmly after the second stage was completed, and showed no tendency to relax afterward.

It diminishes the lochial discharge to a normal standard; many of the patients expressed surprise at the small amount of flow during the twenty-four hours following labor.

Its use is followed by less after-pains than usual in a majority of cases.

It reduces the frequency of the mother's pulse, and relieves the nervous demoralization so often seen in the first stage of labor.

Given during parturition, it never disturs the brain or causes its usual unpleasant effects, even in patients who at other times are very susceptible to its influence. Although the dose has been uniformly fifteen grains, in only one case was the slightest sensation of cinchonism manifest, and that lasting only a moment, in a lady who knew what she had taken and was perhaps quite prepared to feel it.

Finally, I would sum up the conclusions I have adopted, perhaps hastily, though with a certain conviction of their correctness, viz.:

I. That quinia has no inherent property of stimulating the gravid uterus to contration; being inert as to any effect upon the womb in a quiescent state, and having no decided action in accidental labors at any period of gestation.

II. That to its property as a general stimulant and promotor of vital energy and functional activity, and to that alone, is due its influence upon the uterus in normal parturition; producing then no action peculiar to itself, but merely increasing the power of the uterus to expel its contents by its own natural method, converting what is a defective or even pathological action into a simple physiological process.

III. That by availing ourselves of this power, we may, by administering full doses of the sulphate of quinia at the onset of labor, favor the rapid and safe termination of what might otherwise be a tedious and exhausting work.

4. Dr. Thomas, after a few introductory remarks, reports an interesting case of tubal pregnancy which has recently fallen under his observation, and in the treatment of which he met with gratifying success. The patient, a lady aged thirty-two, was a little over three months pregnant when first seen by Dr. Thomas. Up to three weeks previous to the time when first seen she had enjoyed excellent health. During the night of January 15th the patient, who had experienced slight uneasiness in this region previously, was a awakened by a severe "cramp" in the left iliac fossa. This increased to become agonizing in character, and a physician was summoned. The attack was only relieved by the free use of morphia sub-cutaneously. Soreness continued in this region, and at the end of five days another attack similar to the first was experienced. After this the patient was at no time free from pain until seen by Dr. Thomas, on February 4th.

A physical examination revealed the following condition:

The uterus appeared somewhat enlarged to conjoined manipulation, measuring three and a half inches from the os externum to the fundus, was in the position of right oblique anteversion, and was not so movable as normal; the vagina was soft, elastic, and enlarged as it is during pregnancy; to the left of the uterus I found, by vaginal touch, a tense, elastic cyst, which filled the whole iliac fossa, pressed the uterus to the right, and extended downward as low as a point a little below the os internum. Upon conjoined manipulation this cyst was found to be as large as the uterus in the third or third-and-a-half month of pregnancy. It was sensitive to the touch, slightly movable upon upward pressure, and, upon carefully practising *ballottement* upon it, I could feebly but distinctly get the evidence of a very light body which was thrown upward and fell upon the floor of the sac. No doubt existed in my mind as to the recognition of this fact, and it was only after its recognition that I ventured to probe and measure the uterine cavity. Upon it I based the diagnosis which I then made of left tubal pregnancy at the end of the third month of development.

On the 5th of February, one day after my visit, Dr. Marion Sims went out to see the patient; on the 6th Mr. C. called to request me to act as my judgment dictated in reference to the case; and on the 7th I performed the operation, which I now proceed to describe. On Sunday, the 7th, in company with Drs. J. E. Blake, J. B. Hunter, and S. Beach Jones, I went to Elizabeth, prepared to remove the fœtus by elytrotomy. The weather was intensely cold, the thermometer being at zero in Elizabeth. At the house of the patient we were met by Dr. Crane, Dr. Green being detained by a case of midwifery.

Before detailing the treatment of this case, I would remark that few cases of extra-uterine pregnancy have, during their early and progressive stages, been brought to a favorable conclusion by surgical means. For this there are four good reasons: 1. The doubt which usually attends diagnosis; 2. The danger of attending invasion of the peritonæum; 3. The dangers arising from septic absorption from retention of the fœtus or its envelopes; and, 4. The certainty of grave hæmorrhage from opening into the extraordinarily vascular nest in which the fœtus is contained. To meet the indications the following plans have been those generally adopted: 1. The operation of gastrotomy has been practised, that the fœtal mass might be removed like an ovarian tumor; 2. The liquor amnii has been drawn off by a very delicate trocar, in order to diminish tension and check the growth of the cyst; 3. The fœtus has been killed *in situ* by the passage of strong currents of electricity, or the injection into the sac of strong narcotics, like atropia or morphia, with the hope that nature might, at a future time, cause its discharge, with the contents of the abscess which it usually creates.

The first procedure is attended by the dangers of peritonitis and hæmorrhage; and the second and third by those of hæmorrhage into the sac, septicæmia, and subsequent formation and discharge of an abscess located just under the peritonæum. By the process which I now proposed to adopt, I hoped to avoid the dangers of peritonitis by opening the fœtal sac by the vagina, passing up to it between the folds of the broad ligaments. Hæmorrhage, I thought, might be in great degree prevented by cutting into the sac by means of a knife rendered incandescent by a powerful current of electricity. And by complete removal of both fœtus and placenta, and thorough drainage of the sac by carbolized injections through a tube of glass or silver, kept in it, and discharging its contents by the vagina, I was sanguine of avoiding septicæmia.

I proceeded to adopt the plan from which I hoped for these results in the following manner: The patient having been etherized by Dr. Blake, was placed upon a table before a window admitting a strong light, in the left lateral position, and Sims's speculum introduced. Through this cyst to the left of the uterus could be distinctly palpated. Now, fixing a long-handled tenaculum in the cervix uteri, and another in the vagina near the left ilium, this part was by them put on a stretch so as to make that side of the canal a triangle, the base of which was over the cyst, and the apex at the vulva. Assistants held these tenacula during the operation. Taking the platinum knife of the galvano-caustic battery, which was brought to a white heat, I now passed it gently over the base of the triangle described as created in the vagina, carrying it from one tenaculum to the other. By repeating this the vaginal wall, over the lower segment of the cyst, was slowly cut through. In six minutes the cyst was opened by the incandescent knife, and a straw-colored, slightly-pinkish fluid was thrown out with such force as to fly into my face and over my clothing.

Thus far no blood whatever had been lost. I now passed my index finger into the cyst, and felt a fœtus lying horizontally with the head toward the ilium, and the feet toward the uterus. Passing in the middle finger likewise, I caught the feet between the two, and, turning the fœtal body, drew them through the artificial os which I had created, and delivered the child from the vicarious uterus which it occupied. The steps of the procedure exactly resembled those adopted in ordinary podalic version. The fœtal body advanced steadily until the arms reached the opening; then arrest occurred until they were swept out. The head was then arrested, and I strove to liberate it by manipulation and traction. Failing in this, I applied a pair of long-handled placental forceps, and at once it was extracted. The cord was then cut, and I proceeded to deliver the placenta by gentle traction and detachment, as is done after ordinary labor. Thus far thirteen minutes had been consumed.

At this point, the first difficulty which had attended the operation showed itself. When I had separated a little over half of the placenta, a very severe hæmorrhage took place, and so much was the patient's condition depreciated by it in the two or three minutes of its duration, that I was unwilling to delay for the removal of more. Tearing the detached portion off, I passed a large gum-elastic catheter into the sac, and injected a solution of the persulphate of iron into it. This I was very sorry to be forced to do, but the hazard of delay was too great to allow of any other course. The flow of blood was instantly checked, but this was attained at the sacrifice of perfect drainage, and the leaving of the sac full of coagulated blood, and a portion of the placenta. Instead of inserting a drainage-tube, I was forced to substitute a long tent of carbolized cotton, saturated with a solution of persulphate of iron.

In twenty-eight minutes from the commencement of the operation the patient was put to bed, her head kept low, the foot of the bedstead elevated about six inches, ten drops of Magendie's solution of morphia injected subcutaneously, perfect quiet established, and a milk-diet ordered.

After this, all went well until the evening of the fonrth day, when I withdrew the tent of cotton, and symptoms of septicæmia soon showed themselves. These yielded to constantly-repeated injections into the sac of carbolized water, at the end of a week. On the seventh day after the operation, slight hæmorrhage took place from the sac, but was without difficulty controlled by the addition of a small amount of solution of persulphate of iron to the carbolized water.

Six weeks after this operation, I examined by vaginal touch, and was surprised to find the opening made by the incandescent knife so completely closed that I found difficulty in ascertaining its exact location; and ten weeks after it, upon visiting Elizabeth to see another patient, I had the satisfaction of seeing Mrs. C. in her parlor receiving company.

The appreciation of the sign of *ballottement*, which greatly aided me in arriving at a positive diagnosis in this case, has served me an equally valuable purpose in two others. On no sigle sign, however, should undue reliance be placed. During the first sixteen years of my practice, I saw no case of extra-uterine pregnancy. At the end of that period, I saw four in one month. During the past seven years, I have met with nine. Three of these I saw after rupture of the sac, the patients being *in articulo mortis;* two were put beyond question by gradual discharge of fœtal bones; in four cases I succeeded in making a diagnosis as positive as that which is detailed in this paper. In these, a certain conclusion was arrived at by coincidence of the following conditions: (*a.*) The existence of the gastric, mammary, and pelvic symptoms of pregnaucy; (*b.*) An uterus smaller than should exist at the supposed period of gestation; (*c.*) A sensitive tumor to one side of or behind the uterus; and (*d.*) Pains extending from the pelvis down one thigh.

In three of these four cases I so distinctly obtained the sign of *ballottement* as to be willing to lay a good deal of stress upon it in arriving at a decision, and in all of them, having arrived at a conclusion with a good deal of positiveness, I did not hesitate to employ the uterine sound to assure myself of the position, capacity, and state of vacuity of the uterus.

5. The case of Hirsuties is only interesting on account of its rarity and as illustrating the fact that "associated with the pregnant state, and dependent upon its continuance, are numerous manifestations which have their seat in organs so remote that it is difficult, in many cases, to trace the sympathy which exists between them and the special organs of generation."

The patient has borne three children and had one abortion. A peculiarity of each gestation is the growth of a beard on the sides of the face and under the chin. The hairy growth has uniformly started at the commencement of pregnancy and continued until the uterus had resumed its unfecundated condition.

Attention is first called to the parts by a sense of heat and itching which shortly subsides and returns after accouchment and remains until the falling of the hair. The hair is thick-set, firm and soft in texture, straight, and lighter in color than the hair of the head. Its length at childbirth is from one to one and a half inches. Mrs. R. presents no other peculiarity during pregnancy than the hirsute condition, she uniformly has good health. The children present nothing peculiar in appearance.

